# Investigations Into Bioenergetic Neuroprotection of Cone Photoreceptors: Relevance to Retinitis Pigmentosa

**DOI:** 10.3389/fnins.2019.01234

**Published:** 2019-11-15

**Authors:** Daniel S. Narayan, Glyn Chidlow, John P. M. Wood, Robert J. Casson

**Affiliations:** Ophthalmic Research Laboratories, Discipline of Ophthalmology and Visual Sciences, University of Adelaide, Adelaide, SA, Australia

**Keywords:** *rd1* mouse, retinitis pigmentosa, cone photoreceptor, bioenergetic neuroprotection, creatine, nutraceutical, S-opsin, M/L-opsin

## Abstract

Recent studies suggest cone degeneration in retinitis pigmentosa (RP) may result from intracellular energy depletion. We tested the hypothesis that cones die when depleted of energy by examining the effect of two bioenergetic, nutraceutical agents on cone survival. The study had three specific aims: firstly, we, studied the neuroprotective efficacies of glucose and creatine in an *in vitro* model of RP. Next, we utilized a well-characterized mouse model of RP to examine whether surviving cones, devoid of their inner segments, continue to express genes vital for glucose, and creatine utilization. Finally, we analyzed the neuroprotective properties of glucose and creatine on cone photoreceptors in a mouse model of RP. Two different bioenergy-based therapies were tested in *rd1* mice: repeated local delivery of glucose and systemic creatine. Optomotor responses were tested and cone density was quantified on retinal wholemounts. The results showed that glucose supplementation increased survival of cones in culture subjected to mitochondrial stress or oxidative insult. Despite losing their inner segments, surviving cones in the *rd1* retina continued to express the various glycolytic enzymes. Following a single subconjunctival injection, the mean vitreous glucose concentration was significantly elevated at 1 and 8 h, but not at 16 h after injection; however, daily subconjunctival injection of glucose neither enhanced spatial visual performance nor slowed cone cell degeneration in *rd1* mice relative to isotonic saline. Creatine dose-dependently increased survival of cones in culture subjected to mitochondrial dysfunction, but not to oxidative stress. Despite the loss of their mitochondrial-rich inner segments, cone somas and axonal terminals in the *rd1* retina were strongly positive for both the mitochondrial and cytosolic forms of creatine kinase at each time point examined. Creatine-fed *rd1* mice displayed enhanced optomotor responses compared to mice fed normal chow. Moreover, cone density was significantly greater in creatine-treated mice compared to controls. The overall results of this study provide tentative support for the hypothesis that creatine supplementation may delay secondary degeneration of cones in individuals with RP.

## Introduction

Human photoreceptors have a curious and incompletely understood energy metabolism. They derive their nutrient supply from the choriocapillaris, and recent evidence indicates that they are members of a “metabolic ecosystem” comprising the retinal pigment epithelium and adjacent Müller cells (Kanow et al., 2017). Oxidative phosphorylation is an important source of ATP for the retina (Anderson and Saltzman, 1964), and the region of greatest oxygen consumption is the mitochondrial-rich inner segments of the photoreceptors (Cringle et al., 2002). However, mammalian photoreceptors also display aerobic glycolysis (the Warburg effect), producing relatively large amounts of lactate despite the presence of abundant oxygen (Winkler, 1981; Wang et al., 1997; Chinchore et al., 2017; Narayan et al., 2017; Petit et al., 2018). The explanation for this unusual metabolism, which is reminiscent of cancer cells, is unclear, but it seems reasonable to infer that photoreceptors are promiscuous in terms of their energy supply.

Photoreceptors require large amounts of energy to maintain their resting potentials (Ames et al., 1992; Niven et al., 2007), with cones incurring an even greater energy expenditure than rods (Okawa et al., 2008). In the face of this relentless energy demand it seems likely that an impairment of energy metabolism would be detrimental to photoreceptor function with serious consequences for vision. Indeed, there is converging evidence that bioenergetic dysfunction is a key pathogenic factor in secondary cone degeneration in retinitis pigmentosa (RP) (Punzo et al., 2009; Ait-Ali et al., 2015; Narayan et al., 2016; Wang et al., 2016). In the majority of subtypes of RP, the genetic defect is expressed in the rods, but in most individuals the cones eventually degenerate resulting in loss of central vision. Therapeutic targeting of secondary cone degeneration in RP is a broad-spectrum strategy applicable to a large proportion of RP subtypes irrespective of the specific gene defect. Bioenergetic-based neuroprotection strategies, which include augmenting or conserving available energy supplies, boosting mitochondrial efficiency, and improving cellular energy-buffering, and offer great potential in RP. Nutraceutical approaches to bioenergetic neuroprotection gain further appeal by their relative safety and clinical translatability.

Recent studies in animal models of RP have demonstrated that high glucose is critical for cone survival and that reduced glucose entry into cones triggers their degeneration (Punzo et al., 2009; Ait-Ali et al., 2015; Venkatesh et al., 2016). Moreover, a single injection of glucose has been shown to cause a short-term improvement in cone morphology in a slow-progressing porcine model of RP (Wang et al., 2016). We have previously demonstrated that elevating the vitreal glucose level protects retinal ganglion cells against experimental ischemic injury (Casson et al., 2004; Shibeeb et al., 2016) and temporarily recovers contrast sensitivity in individuals with glaucoma (Casson et al., 2014; Shibeeb et al., 2016). Importantly, these effects can be achieved by local delivery of glucose. These insights provide considerable motivation to further investigate glucose energy supplementation as a potential therapy for RP.

A second bioenergetic approach with potential applicability to RP involves boosting levels of the nutraceutical creatine in cones. The creatine kinase/phosphocreatine system, plays an important role in mitochondrial energy metabolism (Wallimann et al., 2011), particularly in cells with high energetic demands such as photoreceptors (Linton et al., 2010). Functions ascribed to the creatine kinase/phosphocreatine kinase system include spatiotemporal buffering of ATP and improving the efficiency of oxidative phosphorylation. The creatine transporter, which is responsible for creatine uptake into cells, and creatine kinase, which catalyzes the reversible conversion of creatine into high energy phosphocreatine, are both expressed in photoreceptors (Acosta et al., 2005; Linton et al., 2010; Rueda et al., 2016; Chidlow et al., 2019); thus, supplementation with creatine is a neuroprotective strategy worthy of investigation.

The present study had three objectives: firstly, we, studied the neuroprotective efficacies of glucose and creatine in an *in vitro* model of RP. Next, we utilized a well-characterized mouse model of RP to examine whether surviving cones, devoid of their inner segments, continue to express genes vital for glucose and creatine utilization. Finally, we analyzed the neuroprotective properties of glucose and creatine on cone photoreceptors in the mouse model of RP.

## Materials and Methods

### Retinal Cells *in vitro*: Establishment, Treatment, and Analysis of Cultures

**FIGURE 1:**
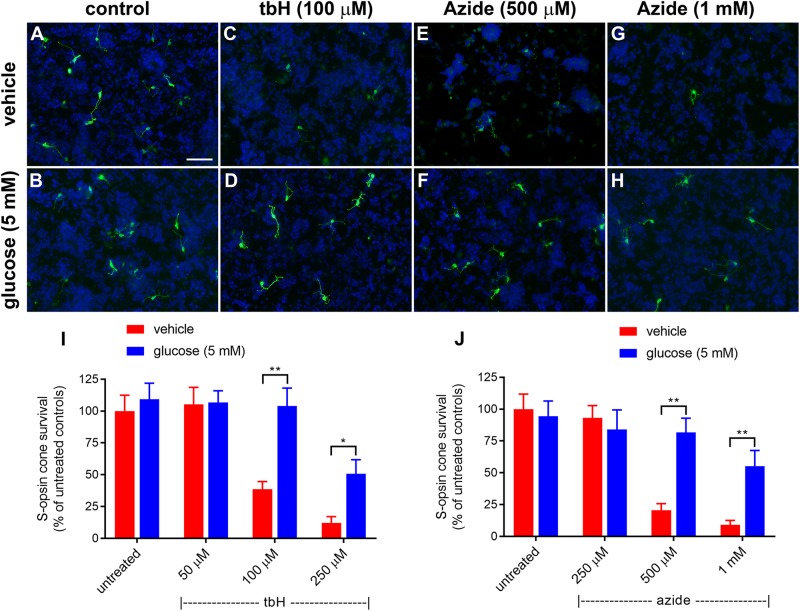
Effect of glucose on stressor-induced S-opsin-labeled cone loss from mixed retinal cell cultures. Representative images from untreated **(A)**, vehicle-treated **(C,E,G)** or glucose (5 mM)-treated **(B,D,F,H)** cultures additionally exposed to **(C,D)**, 100 μM tbH **(E,F)**, 500 μM sodium azide **(G,H)**, 1 mM sodium azide. These data are followed by graphs quantifying the positive effect of glucose on tBH-induced **(I)** and sodium azide-induced **(J)** cone cell loss. It is evident that both tbH and sodium azide are markedly destructive to S-opsin labeled cones in culture and that glucose has clear protective influences on these cells in both cases. Values represent mean ± SEM, where *n* = 8 determinations from separate cultures. ^∗∗^*P* < 0.01, and ^∗^*P* < 0.05 by Student’s paired *t*-test with Holm-Bonferroni correction. Scale bar, 50 μm.

Retinal cultures consisting of a mixed population of cell-types were established from 2 to 4 day old Sprague-Dawley rat pups via a combined enzymatic and mechanical dissociation procedure, as described previously (Wood et al., 2012). Dissociated cells were seeded on borosilicate glass coverslips pre-coated with poly-L-lysine (10 μg/ml; 15 min), at 0.5 × 10^6^ cells/ml, in Minimal Essential Medium (Life Technologies Australia Pty Ltd, Mulgrave, VIC, Australia), containing 100 U/mL penicillin and streptomycin, 2 mM L-glutamine, 5 mM D-glucose and 10% (v/v) fetal bovine serum. Cultures were grown for 6 days *in vitro*, with no medium change during this time. Importantly, immunolabeling of cultures demonstrated the presence of S-opsin-expressing cone photoreceptors (Figure 1), enabling investigation of the responses of these cells to stressors and potential protectants *in vitro*.

Prior to experimental treatments, cultures were changed into an equivalent medium containing no glucose for 24 h; metabolic activity of cells was maintained by the presence of L-glutamine (Wood et al., 2005). For assessment of the potential protective effects of glucose (5 mM) or creatine (0.5 and 5 mM), cultures were pretreated for an additional 24 h with these compounds. To establish oxidative stress, tert-butyl hydroperoxide (tbH; 50 – 250 μM) was applied to cultures for 24 h. To induce metabolic stress, the mitochondrial Complex IV inhibitor sodium azide (250 μM – 1 mM) was applied for 24 h. All stressors and protectants were soluble in cell culture medium and hence, where applied, “vehicle” refers to an added equivalent volume of medium. After treatments, cultures were fixed in 10% (w/v) neutral buffered formalin for 15 min and then processed for cone photoreceptor analysis via immunocytochemistry. A schematic representing the experimental timeline is provided in Supplementary Figure S1.

To assess if sodium azide increased aerobic glycolysis in mixed retinal cultures, cultures were changed into serum-free medium and incubated for 3 h in the presence or absence of sodium azide. Aliquots of medium were then analyzed using a lactate assay kit (BioVision Inc., Milpitas, CA, United States) and absorbances read with a microplate reader (Fluostar Optima; BMG Labtech, Mornington, VIC, Australia).

Survival of cones was assessed by immunocytochemical labeling of fixed cultures with S-opsin antibody (1:1000, see Table 1), as described in the immunohistochemistry methods section below. Nuclear counter-staining of cells was achieved with a final 5 min incubation of coverslips in 500 ng/ml 4′,6-diamidino-2-phenylindole (DAPI) before washing and mounting.

**TABLE 1:** Primary antibodies used in the study.

**Protein**	**Source**	**Cat. No.**	**Species**	**Immunogen**	**Dilution**
CK-B	Abcam	Ab125114	Mouse	Synthetic peptide SNSHNALKLRFPAEDEF, corresponding to N terminal amino acids 4–20 of human creatine kinase B type	1:1000
CK-MT1A	Proteintech	15346-1-AP	Rabbit	CKMT1A fusion protein Ag7583	1:5,000
Hexokinase II	Cell Signaling Technology	2867	Rabbit	Synthetic peptide corresponding to the sequence of human hexokinase II	1:500
LDH-A	Santa-Cruz	sc-27230	Goat	Epitope mapping at the N-terminus of LDH-A of human origin	1:1000
M/L-opsin	Merck-Millipore	AB5405	Rabbit	Recombinant human red/green opsin	1:1500
S-opsin	Santa-Cruz	sc-14363	Goat	Peptide mapping at the N-terminus of the opsin protein encoded by OPN1SW of human origin	1:1500

*LDH-A, lactate dehydrogenase subunit A; CK-B, creatine kinase B-type; CK-MT1A, creatine kinase mitochondrial 1A.*

### Animals and Procedures

This study was approved by the Animal Ethics Committees of SA Pathology/Central Adelaide Local Health Network (CALHN) and the University of Adelaide (Adelaide, SA, Australia) and conformed with the Australian Code of Practice for the Care and Use of Animals for Scientific Purposes, 2013, and with the ARVO Statement for the use of animals in vision and ophthalmic research. C3H/HeJArc (*rd1*) mice and C57BL/6J (wild-type, WT) mice were obtained from the Animal Resources Centre (Perth, WA, Australia). The C3H/HeJArc mouse is an inbred strain homozygous for the PDE6b mutation. *rd1* mice were inbred to produce offspring. All animals were housed in a temperature and humidity-controlled room with a 12-h light/dark cycle and provided with food and water *ad libitum*.

For detection of cone opsin proteins, creatine kinase isoforms, and glycolytic enzymes by immunohistochemistry, *rd1* mice at various postnatal day (P) time points [P7, P14, P21, P30, P45, and P60 (all *n* = 3) and WT mice (C57BL/6, *n* = 3)] were analyzed.

For the glucose neuroprotection study, *rd1* mice were randomly divided into experimental (*n* = 13) and control (*n* = 11) groups. At the starting age of P14, the right eyes of experimental mice were treated with an injection of 20 μl of 50% glucose into the subconjunctival space (see below). Injections were repeated at 24-hourly intervals. The left eye remained as an untreated control. Mice in the control group received a subconjunctival injection of osmotically matched 8.4% saline. Again, the fellow eye served as an untreated control. Injections were continued for 30 days in both groups. Optomotor testing was performed 10 days into the treatment regimen at P24. Mice were euthanized at P60.

For the creatine neuroprotection study, *rd1* mice were randomly divided into experimental (*n* = 21) and control (*n* = 18) groups. The experimental group was placed on a 2% oral creatine diet starting at the age of P21. Mice litters were weaned from their parents at P21 and this was the age oral treatment with creatine was commenced. Control animals were given an equicaloric standard mouse diet, without creatine. Mice were carefully observed to monitor their eating. Because the creatine was incorporated in the food, and mice were kept at 4 – 5 per cage, it was difficult to accurately assess how much food each mouse consumed. Daily weighing of mice was performed, and satisfactory weight gain was used as an indicator of appropriate food consumption. Optomotor testing was performed at P30. Mice in experimental and control groups continued their diet until they were euthanized at P60.

### Tissue Processing and Immunohistochemistry

All mice were euthanized by transcardial perfusion with physiological saline under deep anesthesia. The superior aspect of each cornea was marked for orientation before globes were enucleated. For double labeling wholemount immunohistochemistry, eyes were fixed in 4% (w/v) neutral buffered formalin for 24 h and dissected into posterior eye-cups. Retinas were removed and prepared as flattened wholemounts by making another four radial cuts. Retinas were then incubated in phosphate buffered saline (PBS) containing 1% Triton X-100 detergent (PBS-T) for 1 h at room temperature. Next, retinas were incubated in PBS-T containing 3% normal horse serum (NHS-T) for 1 h at room temperature to block non-specific antibody binding. Retinas were then incubated overnight at 4°C with a combination of anti-S-opsin and anti-M/L-opsin antibodies diluted in NHS-T (see Table 1). On day 2, retinas were washed for 1 h at room temperature in PBS-T, then incubated overnight at 4°C with a combination of AlexaFluor-488 and -594 conjugated secondary antibodies (1:250; Invitrogen, Carlsbad, CA, United States) diluted in NHS-T. Finally, retinas were washed in PBS for 1 h at room temperature prior to mounting with the photoreceptor side facing up using anti-fade mounting medium (Dako, CA, United States).

For immunohistochemistry on transverse sections, globes were immersion-fixed in Davidson’s solution for 24 h and transferred to 70% ethanol until processing. Davidson’s solution, which comprises 2 parts formaldehyde (37%), 3 parts 100% ethanol, 1 part glacial acetic acid and 3 parts water, is the preferred fixative for whole eyes as it provides optimal tissue morphology while avoiding retinal detachment. Globes were embedded sagittally and 4 μm serial sections were cut. For fluorescent double labeling immunohistochemistry, visualization of one antigen was achieved using a 3-step procedure, while the second antigen was labeled by a 2-step procedure, as previously described (Chidlow et al., 2011, 2016). In brief, tissue sections were deparaffinized and high-temperature antigen retrieval was performed. Subsequently, sections were incubated overnight in the appropriate combination of primary antibodies (Table 1). On the following day, sections were incubated with the appropriate biotinylated secondary antibody (1:250) for the 3-step procedure plus the correct secondary antibody conjugated to AlexaFluor 488 (1:250; Invitrogen) for the 2-step procedure for 30 min, followed by streptavidin-conjugated AlexaFluor 594 (1:500; Invitrogen) for 1 h. Sections were then mounted using anti-fade mounting medium.

### Image Acquisition and Quantification

Confirmation of the specificity of antibody labeling was judged by the morphology and distribution of the labeled cells, by the absence of signal when the primary antibody was replaced by isotype/serum controls, and, by comparison with the expected staining pattern based on our own, and other, previously published results. Retinal wholemounts and transverse sections were examined under a fluorescence microscope (BX-61; Olympus, Mount Waverly, VIC, Australia) equipped with a scientific grade, cooled CCD camera.

Photomicrographs measuring 720 μm x 540 μm were taken using the fluorescence microscope on whole mounts retinas. Four photomicrographs of the central retina were taken directly superior, temporal, inferior, and nasal to the center of the optic nerve. Four photomicrographs of the peripheral retina were taken 1.5 mm superior, temporal, inferior, and nasal to the center of the optic nerve. This yielded a total of 16 photomicrographs per retina. Quantification of cone survival was performed using Image-J software (NIH, Bethesda, MD, United States). Initially, however, images were processed in Photoshop CS3 (Adobe, San Jose, CA, United States) for uneven lighting (using a flatten filter), sharpened, levels enhanced, and finally converted to 8-bit mode.

For the glucose experiment, three statistical comparisons were performed: glucose-injected vs. untreated contralateral eyes (Student’s paired *t*-test); saline-injected vs. untreated contralateral eyes (Student’s paired *t*-test); saline-injected eyes vs. glucose-injected eyes (Student’s unpaired *t*-test). For the creatine experiment, statistical analysis was carried out by Student’s unpaired *t*-test. The null hypothesis tested in each case was that cone density would be significantly higher in experimental vs. control eyes.

### Measurement of Vitreal and Retinal Glucose After Subconjunctival Injection

Subconjunctival drug administration bypasses the conjunctival epithelial barrier, which is a rate-limiting factor for the permeation of water-soluble drugs (Gaudana et al., 2010); however, various dynamic, static, and metabolic barriers limit drug access to the posterior segment and retina. In order to establish whether subconjunctival glucose reaches the retina, a preliminary investigation was performed to measure the vitreous glucose concentration after subconjunctival injection. A single injection of 20 μl of 50% glucose was administered into the subconjunctival space in the right eyes of C57BL/6J mice. This procedure involved lifting the inside of the eyelid with fine forceps and delivering a subconjunctival injection using a 33G needle. Use of an operating microscope facilitated the procedure. Injections were performed under isoflurane sedation and topical amethocaine 0.5% anesthetic. During every injection, visualization of a subconjunctival bleb provided confirmation that the injection had been successfully administered. Left eyes were treated with an injection of osmotically matched 8.4% saline. Mice were euthanized after 1, 8, and 16 h (*n* = 3 per time point). Eyes were enucleated, the vitreous was removed as described previously (Skeie et al., 2011) and then sonicated. Vitreal samples (diluted to a final volume of 100 μl with distilled water) were then analyzed using a glucose hexokinase assay kit (Sigma-Aldrich Corp., St Louis, MO, United States) and absorbances read with a microplate reader (Fluostar Optima). The final vitreous glucose concentration (mmol/L) was determined by allowing for the 10-fold dilution factor, and comparing the absorbance with a previously constructed calibration curve. Statistical analysis was carried out by Student’s paired *t*-test with Holm-Bonferroni correction. The null hypothesis tested was that the vitreous glucose concentration would be significantly higher in glucose- vs. saline-injected eyes.

A further experiment (*n* = 3) was carried out in which both the vitreal and retinal concentration of glucose was measured 30 min after a single subconjunctival injection of 20 μl of 50% glucose, using the method detailed above.

To analyze retinal uptake of 2-NBDG, mice underwent a single subconjunctival injection of 20 μl of 10 mM 2-NBDG (Invitrogen, Carlsbad, CA, United States) dissolved in water. After 30 min, mice were euthanized by exposure to a rising concentration of CO_2_ and globes were enucleated under red light. They were then snap frozen in dry ice-cooled isopentane. Frozen, vertical sections (10 μm) were taken, mounted using anti-fade mounting medium, and examined under a fluorescence microscope.

### Optomotor Testing

This test measures the tendency of an animal to follow a moving object using their head and eyes. Optomotor testing is an established method for quantifying visual function in mice (Prusky et al., 2004). The room was darkened for testing. The only illumination came from the LED screens of the optomotor cage. However, animals were light adapted before starting the test, i.e., they were tested under photopic conditions. Mice were placed on a platform positioned in the middle of an arena created by a quad square of computer monitors. Vertical sine wave gratings (100% contrast) were projected on the computer monitors. The spatial frequencies tested were 0.05, 0.075, 0.1, 0.2, 0.3, 0.4, 0.5, and 0.6 cycles per degree, at a constant speed of 12 degrees/s. A camera was positioned directly above the platform in order to observe the animal’s head movements. Mice were placed one at a time on the platform and allowed to move freely while the experimenter followed the mouse’s head. When a grating perceptible to the mouse was projected on the cylinder wall and the cylinder was rotated (12 degrees/s), the mouse normally stopped moving its body and would begin to track the grating with reflexive head movements in concert with the rotation. A recorder assessed whether the mice tracked the cylinder by monitoring in the video window the image of the cylinder and the animal simultaneously. The spatial frequency of the grating was systematically increased the until the animal no longer responded. The process of incrementally changing the spatial frequency of the test grating was repeated until the highest spatial frequency that the mouse could track was identified as the threshold. A threshold for each direction of rotation was assessed this way, and the highest spatial frequency tracked in either direction was recorded as the threshold. Recorders were blinded to the treatment group and all mice were habituated before the outset of testing. For the glucose experiment, three statistical comparisons were performed: glucose-injected vs. untreated contralateral eyes (Student’s paired *t*-test); saline-injected vs. untreated contralateral eyes (Student’s paired *t*-test); saline-injected eyes vs. glucose-injected eyes (Student’s unpaired *t*-test). For the creatine experiment, statistical analysis was carried out by Student’s unpaired *t*-test. The null hypothesis tested in each case was that visual function would be significantly greater in experimental vs. control eyes.

## Results

### Glucose Supplementation Protects Cones in Culture From Mitochondrial Dysfunction and Oxidative Injury

In order to investigate responses and potential protection of cone cells in culture, they were treated with one of two stressors: (1) sodium azide, which in inhibiting mitochondrial Complex IV causes metabolic compromise and (2) tbH, which induces intracellular oxidative stress. Both stressors mimic aspects of the pathology believed to be associated with RP (Narayan et al., 2016). Of note, sodium azide, by reducing mitochondrial activity, causes a compensatory increase in glycolysis, which is evidenced by increased lactate in the culture medium (Supplementary Figure S1).

Initially, we tested the protective effects of enriching the culture medium with 5 mM glucose; data are shown in Figure 1. Treatment with tbH caused a concentration-dependent loss of S-opsin cones with no observed decrease when 50 μM was applied, but with only 38.7 ± 5.9% or 12.0 ± 5.1% of cells remaining, respectively, when 100 μM or 250 μM tbH were applied. The presence of 5 mM glucose in the medium for 24 h prior to application of tbH was significantly protective: 104.0 ± 14.1% and 50.1 ± 11.1% of the control cell number remained when cultures were treated with 100 μM and 250 μM tbH, respectively, in the presence of glucose (Figure 1).

Mitochondrial dysfunction, as induced by treatment of cultures with sodium azide for 24 h, caused a concentration-dependent loss of cones, with no significant loss when 250 μM was applied (93.1 ± 9.6% of control value), but significant decreases noted when 500 μM (20.7 ± 5.0% of control) or 1 mM (9.2 ± 4.0% of control) were applied (Figure 1). Pre-treatment with glucose significantly reduced the loss of cones with 81.6 ± 11.3% and 55.2 ± 12.3% of cones remaining after treatment with 500 μM and 1 mM azide, respectively.

### Subconjunctival Delivery of Glucose Elevates the Vitreous Glucose Concentration

**FIGURE 2:**
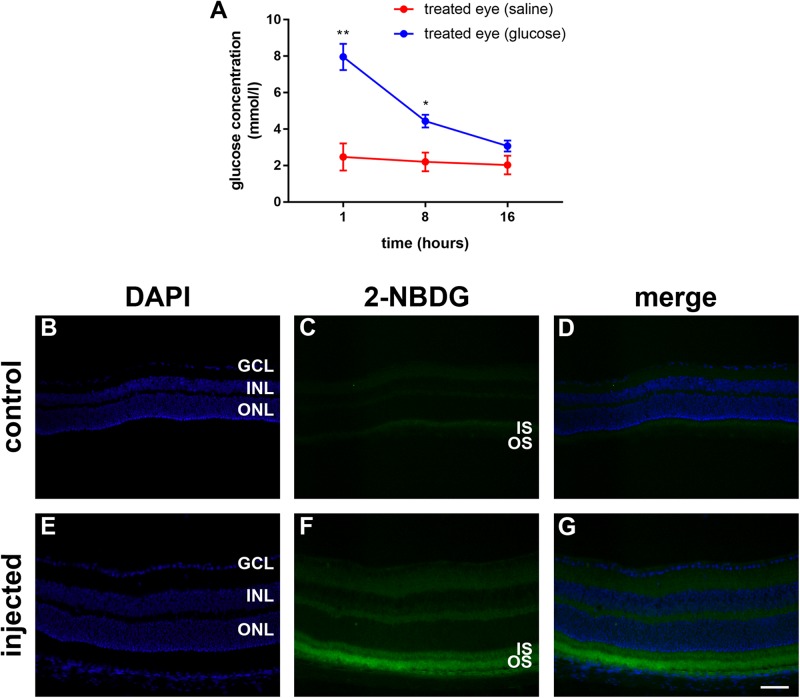
**(A)** Quantification of the vitreous glucose concentration in C57BL/6J mice at 1 to 16 h after subconjunctival injection of either glucose or saline. Values represent mean ± SEM, where *n* = 3. ^∗∗^*P* < 0.01, ^∗^*P* < 0.05 by Student’s paired *t*-test with Holm-Bonferroni correction. **(B–G)** 2-NBDG accumulation in C57BL/6J mice retinas 20 min after subconjunctival injection. In the control mouse, minimal fluorescence is observed **(B–D)**. In the 2-NBDG injected eye, fluorescent glucose accumulates in photoreceptor segments and, to a lesser extent, in both plexiform layers **(E–G)**. Scale bar: 50 μm. GCL, ganglion cell layer; INL, inner nuclear layer; IS, inner segments; ONL, outer nuclear layer; and OS, outer segments.

Prior to examining whether daily injections of glucose delay cone degeneration in a mouse model of RP, it was important to ascertain whether the delivery strategy resulted in a detectable increase in the level of glucose available to the retina. Thus, we measured the vitreous glucose concentration at time points after single subconjunctival injections of glucose or osmotically matched saline in WT mice. The results revealed statistically significant upregulations of glucose in the treated eye, compared to the saline-injected contralateral eye, at 1 h (*P* < 0.01, by paired Student’s *t*-test) and 8 h (*P* < 0.05), but not 16 h, after injection (Figure 2A). Next, we part-repeated the experiment, except on this occasion we measured the retinal, as well as vitreal, glucose concentration at 1 h after injection, the time point of maximal elevation. In the vitreous, the glucose injected eye contained 9.8 ± 0.7 mmol/l glucose, which was significantly (*P* < 0.01 by Student’s unpaired *t*-test) higher than the untreated contralateral eye (2.3 ± 0.2). In the retina, the glucose injected eye contained 38.3 ± 14.4 μg/retina glucose, whilst the untreated contralateral eye contained 16.0 ± 6.7 μg/retina glucose, a difference that did not reach statistical significance (*P* > 0.1). Of note, it is not possible for us to rule out that each retinal sample was contaminated by vitreous. In the mouse, the vitreous adheres to the retina tightly and this may have contributed to the trend of an elevated concentration in the retinal sample of the glucose injected eye.

In order to ascertain whether glucose reaches the photoreceptors after subconjunctival injection, we utilized 2-deoxy glucose (2-NDBG), a stable, fluorescent glucose derivative used for monitoring glucose uptake into living cells (Kanow et al., 2017). At 30 min after subconjunctival injection of 10 mM 2-NDBG, we harvested the retina for tissue sectioning and fluorescent imaging. Figures 2B–G reveals that 2-NBDG fluorescence was weakly localized to the inner and outer plexiform layers and was stronger in the photoreceptor inner and outer segments, confirming that glucose reaches the photoreceptor layer after subconjunctival injection.

### Surviving Cones in the *rd1* Retina Express Glycolytic Enzymes

Photoreceptors have a high glycolytic flux and rate of lactate production even in the presence of oxygen, a phenomenon known as aerobic glycolysis. Accordingly, photoreceptors express glycolytic isoenzymes associated with aerobic glycolysis, including hexokinase II, pyruvate kinase M2 and lactate dehydrogenase (LDH). An increasing body of evidence suggests that energy starvation contributes to secondary degeneration of cones in RP (Punzo et al., 2009; Petit et al., 2018). Since glycolytic enzymes are concentrated in photoreceptor inner segments, and since loss of cone segments is an early pathological event in RP, we investigated whether surviving cones in the *rd1* retina continue to express genes vital for glucose utilization, including hexokinase II and lactate dehydrogenase subunit A (LDH-A).

**FIGURE 3:**
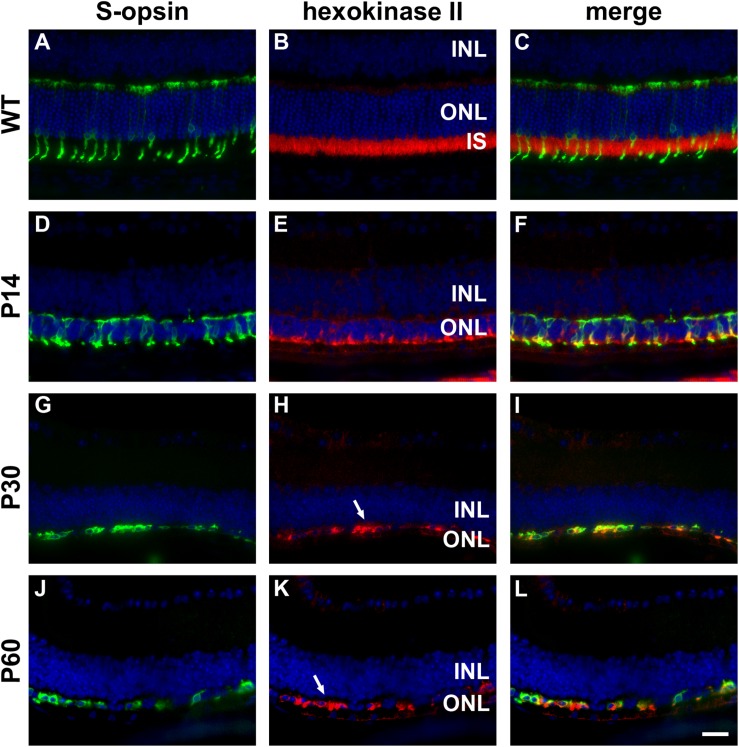
Representative double labeling immunofluorescence images of hexokinase II with S-opsin in wild-type (WT) mouse retina and in *Rd1* mouse retinas from postnatal day (P) 14 to P60. **(A–C)** In WT retinas, hexokinase II is essentially restricted to rod and cone photoreceptor inner segments. **(D–F)** At P14, hexokinase II colocalizes with S-opsin in shrunken inner segments (arrow) and weakly labels occasional cone somas. **(G–L)** From P30 to P60, the ONL comprises a single layer of surviving cones largely devoid of segments. Hexokinase II colocalizes with S-opsin in cone somas (arrows). All images were captured from the inferior retina, which comprises primarily S-cones. INL, inner nuclear layer; IS, inner segments, ONL, outer nuclear layer; RPE, retinal pigment epithelium. Scale bar: 20 μm.

In WT retinas, hexokinase II labeling was essentially restricted to rod and cone photoreceptor inner segments (Figures 3A–C). Despite the loss of inner segments, surviving cones in the *rd1* retina were strongly positive for hexokinase II at all time points examined, encompassing P7 to P60 (see Figures 3D–L for representative images).

**FIGURE 4:**
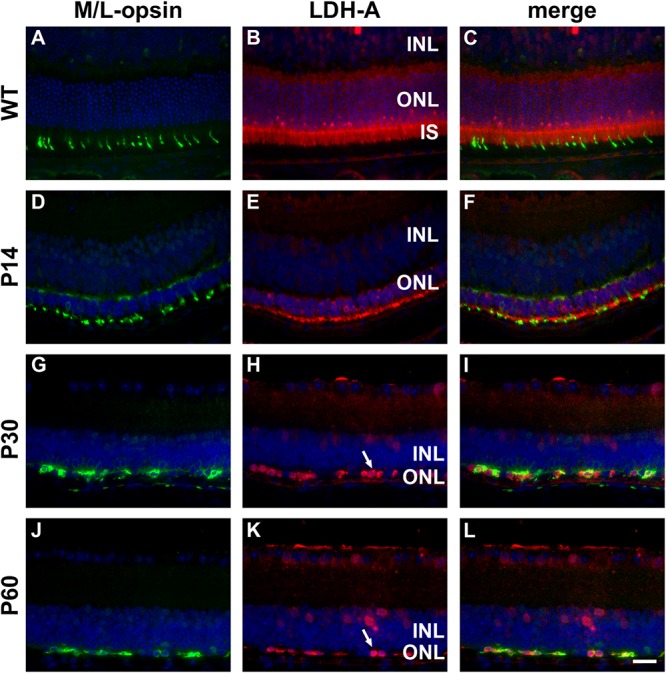
Representative double labeling immunofluorescence images of LDH-A with M/L-opsin in wild-type (WT) mouse retina and in *Rd1* mouse retinas from postnatal day (P) 14 to P60. **(A–C)** In WT retinas, LDH-A is associated with rod and cone photoreceptor somas, inner segments and axonal terminals, together with cells in the inner retina. **(D–F)** At P14, hexokinase II colocalizes with M/L-opsin in shrunken inner segments (arrow) and weakly labels photoreceptor somas and axonal terminals. **(G–L)** From P30 to P60, the ONL comprises a single layer of surviving cones largely devoid of segments. LDH-A is observed in surviving cone somas (arrows). All images were captured from the superior retina, which comprises primarily M/L-cones. INL, inner nuclear layer; IS, inner segments, ONL, outer nuclear layer; RPE, retinal pigment epithelium. Scale bar: 20 μm.

In WT retinas, LDH-A was expressed by rod and cone photoreceptor somas, inner segments and axonal terminals, plus a limited number of cells in the inner retina (Figures 4A–C). In the *rd1* retina, LDH-A labeling was unequivocally expressed by surviving cones at each time point examined, encompassing P7 to P60 (see Figures 4D–L for representative images).

In addition to hexokinase II and LDH-A, surviving cones also displayed PKM2 and neuron-specific enolase immunoreactivities (data not shown). The overall data indicate that despite losing their inner segments, surviving cones in the *rd1* retina retain their complement of enzymes necessary to metabolize glucose.

### Subconjunctival Delivery of Glucose Does Not Preserve Cones in *rd1* Retinas

**FIGURE 5:**
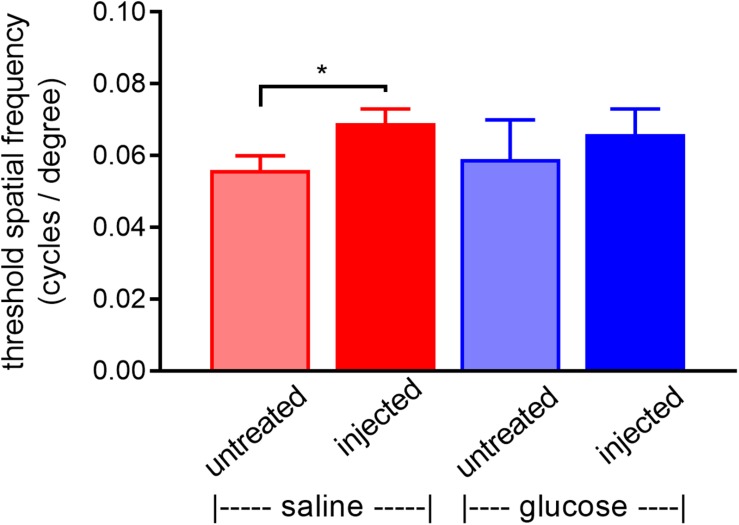
Performance of *Rd1* mice in the optomotor tracking test undertaken at P24, following 10 days of treatment. *Rd1* mice received a daily unilateral subconjunctival injection of glucose or saline; the respective contralateral eyes were untreated. Values represent mean ± SEM, where *n* = 11–13. ^∗^*P* < 0.05 by Student’s paired *t*-test (saline-injected vs. untreated contralateral eyes). There were no significant differences between glucose-injected and untreated contralateral eyes (Student’s paired *t*-test) or between saline-injected eyes and glucose-injected eyes (Student’s unpaired *t*-test).

*rd1* mice received a daily unilateral injection of glucose or osmotically matched saline for 30 days. After 10 days of injections, their visual acuity was assessed using the optomotor reflex test. Both the saline-injected and glucose-injected groups displayed modestly enhanced optomotor responses compared to their respective contralateral eyes (Figure 5), although only the saline-injected group reached statistical significance (*P* < 0.01, by paired Student’s *t*-test). Importantly, there was no difference between the glucose-injected and saline-injected groups (*P* = 0.25, by unpaired Student’s *t*-test).

**FIGURE 6:**
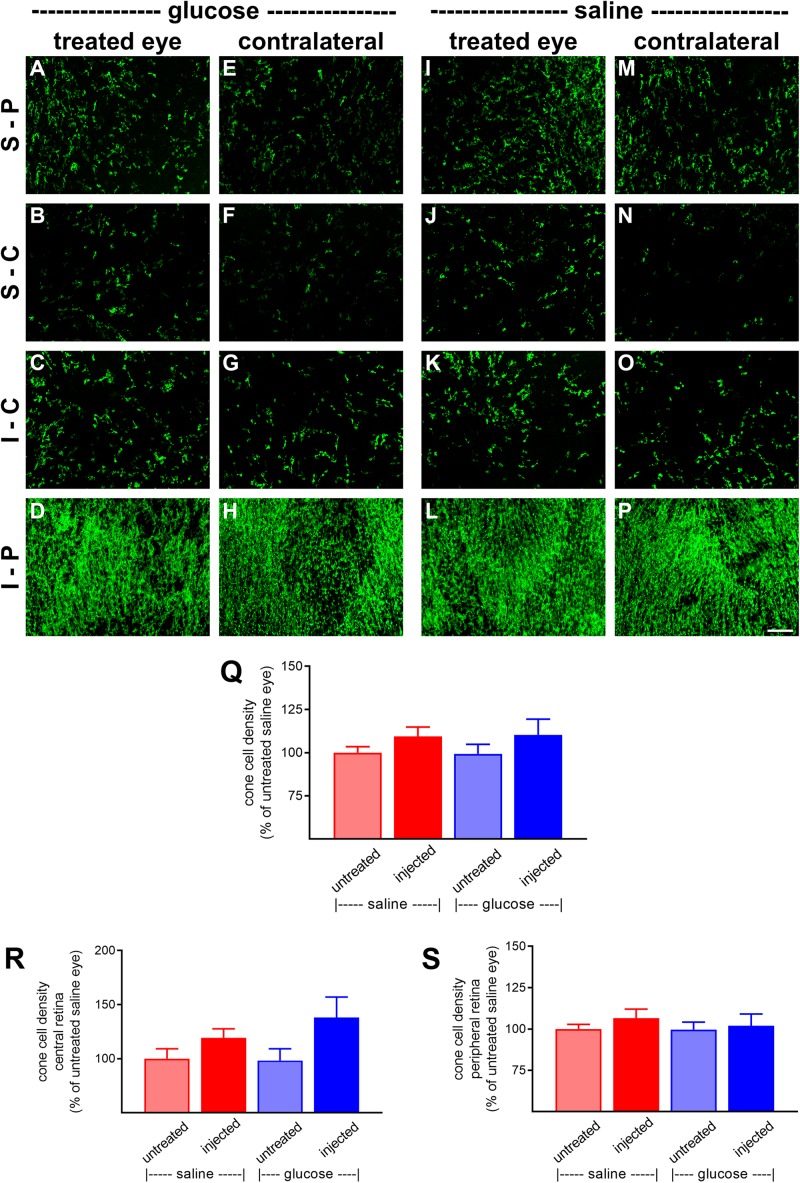
Effect of daily glucose injections on S-opsin^+^-cone survival. **(A–P)** Photomicrographs of wholemount *Rd1* retinas analyzed at postnatal day (P) 60 immunolabeled for S-opsin. Representative images from glucose-injected eyes **(A–D)** and the respective contralateral untreated eyes **(E–H)**, saline-injected eyes **(I–L),** and the respective contralateral untreated eyes **(M–P)** are shown. S-P, superior-peripheral retina; S-C, superior-central retina; I-C, inferior-central retina; I-P, inferior-peripheral retina. Scale bar 100 μm. **(Q–S)** Quantification of S-opsin-labeled cones in retinal wholemounts from *Rd1* mice treated with daily unilateral subconjunctival injections of glucose or saline, and in the respective untreated contralateral eyes. Data are shown for the whole retina **(Q)**, subdivided into central **(R)**, and peripheral **(S)** regions. Values, shown as % of the untreated saline group, represent mean ± SEM, where *n* = 11–13. There were no significant differences between glucose-injected and untreated contralateral eyes (Student’s paired *t*-test), saline-injected vs. untreated contralateral eyes (Student’s paired *t*-test), or between saline-injected eyes and glucose-injected eyes (Student’s unpaired *t*-test).

**FIGURE 7:**
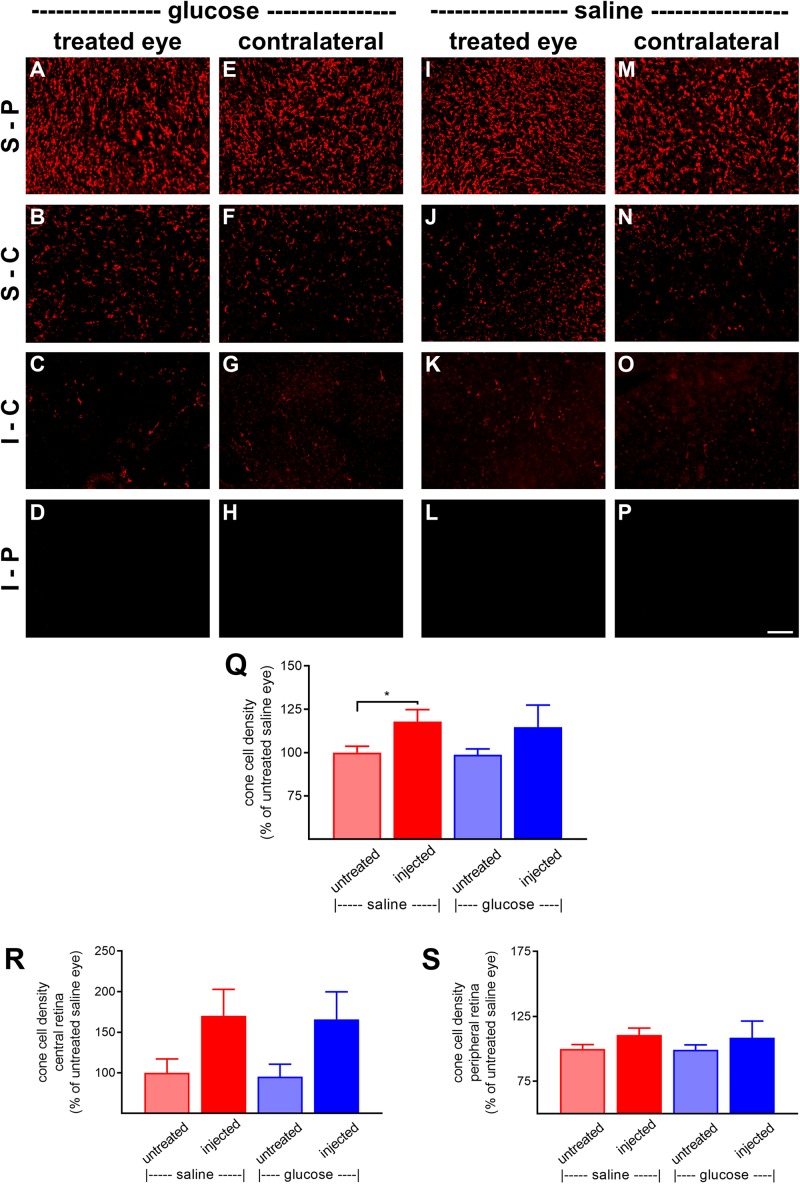
Effect of daily glucose injections on M/L-opsin^+^-cone survival. **(A–P)** Photomicrographs of wholemount *Rd1* retinas analyzed at postnatal day (P) 60 immunolabeled for M/L-opsin. Representative images from glucose-injected eyes **(A–D)** and the respective contralateral untreated eyes **(E–H)**, saline-injected eyes **(I–L)** and the respective contralateral untreated eyes **(M–P)** are shown. S-P, superior-peripheral retina; S-C, superior-central retina; I-C, inferior-central retina; I-P, inferior-peripheral retina. Scale bar 100 μm. **(Q–S)** Quantification of S-opsin-labeled cones in retinal wholemounts from *Rd1* mice treated with daily unilateral subconjunctival injections of glucose or saline, and in the respective untreated contralateral eyes. Data are shown for the whole retina **(Q),** subdivided into central **(R)**, and peripheral **(S)** regions. Values, shown as % of the untreated saline group, represent mean ± SEM, where *n* = 11–13. ^∗^*P* < 0.05 by Student’s paired *t*-test (saline-injected vs. untreated contralateral eyes). There were no significant differences between glucose-injected and untreated contralateral eyes (Student’s paired *t*-test) or between saline-injected eyes and glucose-injected eyes (Student’s unpaired *t*-test).

At P60, the number of surviving cones were quantified. Mouse retinas contain three types of cones, short wavelength light-responsive S-cones, medium to long wavelength light-responsive M/L-cones, and a majority of dual cones that express both opsins (Applebury et al., 2000; Haverkamp et al., 2005; Ortin-Martinez et al., 2014). In the *rd1* mouse, degeneration of cones is more rapid in the central than peripheral retina (Carter-Dawson et al., 1978; Lin et al., 2009). Figures 6A–P, 7A–P, respectively, feature representative images of S-opsin^+^ and M/L-opsin^+^ cones from the different treatment groups. The photomicrographs highlight the central to peripheral divergence in cone survival in the *rd1* retina. The photomicrographs also reveal that, at P60, M/L-opsin^+^ cones are more numerous in the superior retina relative to the inferior retina, whereas S-opsin^+^ cones are far more numerous in the inferior retina. This hemispheric asymmetry in S-opsin^+^ and M/L-opsin^+^ cone expression is in agreement with earlier findings (Lin et al., 2009; Narayan et al., 2019). The very low numbers of M/L-opsin^+^ cones in the inferior retina does not necessarily indicate that dual cones are preferentially lost in the inferior retina, instead it likely reflects the fact that M/L-opsin protein expression is intrinsically very much lower in cones in the inferior retina as compared to the superior retina (Applebury et al., 2000; Haverkamp et al., 2005). With the loss of their outer segments – which contain extraordinarily high levels of opsins – M/L-opsin is no longer detectable in dual cone somas and pedicles, which appear as apparent S-cones. The low numbers of S-opsin^+^ cones in the superior retina simply reflects the fact that there are far fewer S-opsin^+^ cones in the superior as compared to inferior retina. Quantification of the density of S-opsin^+^ and M/L-opsin^+^ cones in the whole retina showed a trend of higher counts of both cone types in each of the saline-injected and glucose-injected groups when compared to their respective, paired, contralateral eyes (Figures 6Q, 7Q), although only the saline-injected group M/L-opsin^+^ cone count reached statistical significance (*P* < 0.05, by paired Student’s *t*-test). As for the optomotor testing, there was no significant difference between the number of S-opsin^+^ cones or M/L-opsin^+^ cones in the glucose-injected vs. saline-injected cohorts (*P* = 0.25, by unpaired Student’s *t*-test). When the data were subdivided into central (Figures 6R, 7R) and peripheral (Figures 6S, 7S) regions, the same patterns of cone survival were evident, namely a non-significant trend of higher counts of both cone types in each of the saline-injected and glucose-injected groups when compared to their respective contralateral eyes. As for the pan retinal quantification, there was no significant difference between the glucose-injected vs. saline-injected cohorts in survival of either cone type in the central or peripheral retina regions of the retina.

### Creatine Supplementation Protects Cones in Culture From Mitochondrial Dysfunction but Not Oxidative Injury

**FIGURE 8:**
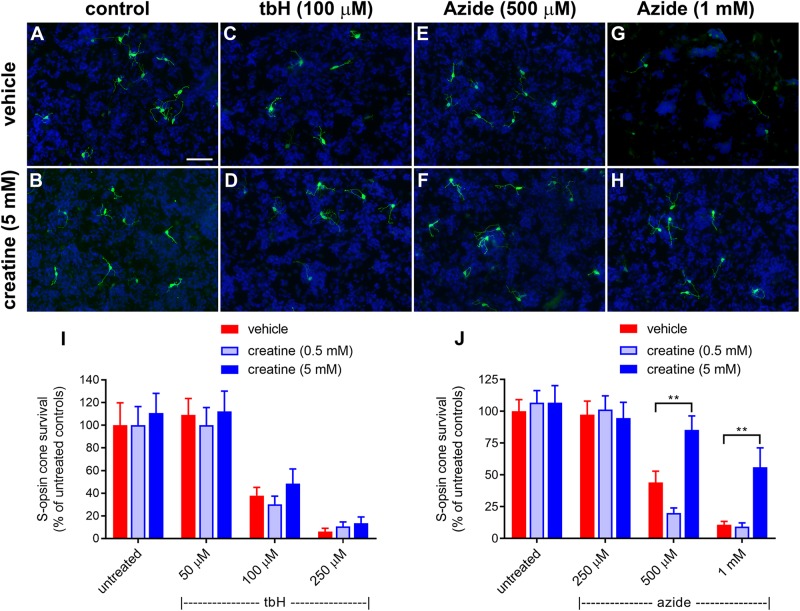
Effect of creatine on stressor-induced S-opsin-labeled cone loss from mixed retinal cell cultures. Representative images from untreated **(A)**, vehicle-treated **(C,E,G)** or creatine (5 mM)-treated **(B,D,F,H)** cultures additionally exposed to **(C,D)**, 100 μM tbH **(E,F)**, 500 μM sodium azide **(G,H)**, 1 mM sodium azide. These data are followed by graphs quantifying the effect of creatine on tBH-induced **(I)** and sodium azide-induced **(J)** cone cell loss. It is evident that both tbH and sodium azide cause marked loss of S-opsin labeled cones in culture. It is further clear that although creatine has no effect on the cone loss induced by tbH, it is able to significantly protect these cells from sodium azide-induced toxicity. Values represent mean ± SEM, where *n* = 8 determinations from separate cultures. ^∗∗^*P* < 0.01, by one way ANOVA, followed by Dunnett’s multiple comparisons test. Scale bar, 50 μm.

The effect of a creatine on survival of cones after induction of stress *in vitro* was assessed after a 24-h pre-treatment. Similar to the glucose experiments shown in Figure 1, tbH application caused a reduction to 37.9 ± 7.2% and 6.0 ± 3.0% of the control cell number when applied at 100 μM or 250 μM, respectively (Figure 8). Creatine pre-treatment (0.5 or 5 mM) had no significant effect on promoting additional survival of cells treated with tbH.

Sodium azide also caused a concentration-dependent loss of S-opsin-labeling cones from mixed retinal cell cultures: 44 ± 8.9% and 10.7 ± 2.7% of the control cell number of cones remained after application of 500 μM and 1 mM sodium azide, respectively (Figure 8). In this case, pre-treatment with creatine at the concentration of 0.5 mM was also not protective to cells, however, when applied at 5 mM, this compound did offer significant protection: there were 85.3 ± 11.0% and 56.0 ± 15.2% of the control number of cells remaining after treatment with 500 μM and 1 mM sodium azide, respectively (Figure 8).

### Surviving Cones in the *rd1* Retina Express Creatine Kinase Isoenzymes

The creatine kinase/phosphocreatine system functions as a spatial and temporal energy buffer in cells, linking sites of energy production, and utilization. Typically, mitochondrial creatine kinase (MT-CK1A) converts creatine to high energy phosphocreatine at sites of ATP production, generating a diffusible energy substrate, whilst the cytoplasmic isoform (CK-B) catalyzes the reverse reaction at subcellular locations of energy usage (Wallimann et al., 2011). Before investigating whether augmenting retinal creatine delays cone loss in the *rd1* retina, it was important to ascertain whether the enzymes of the creatine-phosphocreatine system are preserved in surviving cones of the *rd1* retina during the period of cone degeneration. This is important since mitochondrial creatine kinase is principally localized in photoreceptor inner segments.

**FIGURE 9:**
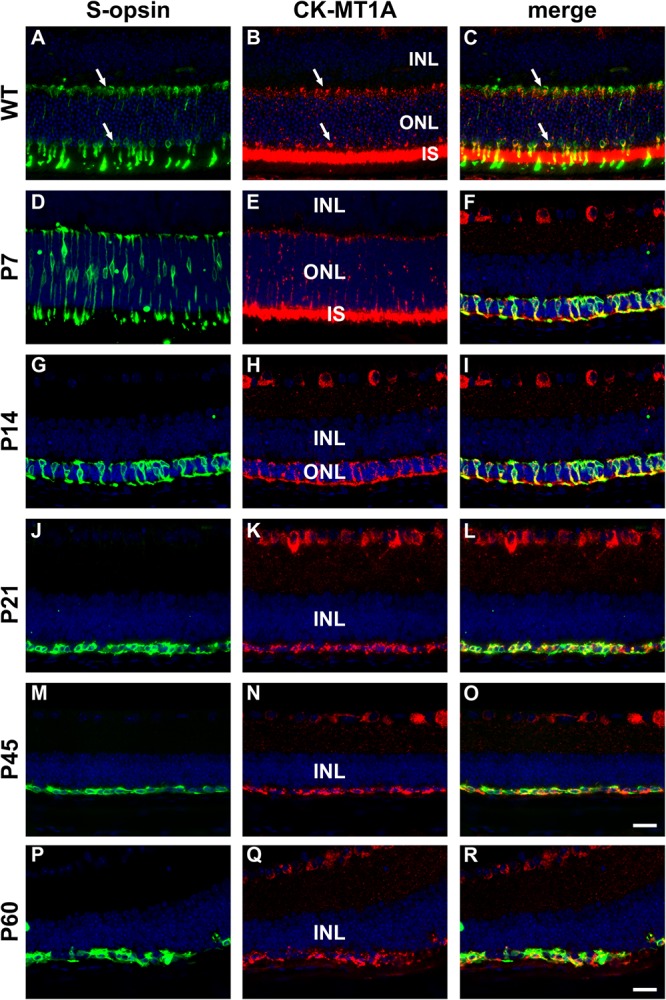
Representative double labeling immunofluorescence images of ubiquitous mitochondrial creatine kinase (CK-MT1A) with S-opsin in wild-type mouse retina and *Rd1* mouse retinas from postnatal day (P) 7 to P60. In WT retinas **(A–C)** and at P7 **(D–F)**, CK-MT1A colocalizes with S-opsin in photoreceptor inner segments, cone somas, and axonal terminals. At P14, the ONL is dramatically reduced in thickness. CK-MT1A colocalizes with S-opsin in shrunken inner segments, cone somas, and axonal terminals **(G–I)**. From P21 onward, the ONL is reduced to a single layer of surviving cones largely devoid of segments. CK-MT1A expression within S-opsin-positive cone somas and axonal terminals persists at all time points **(J–R)**. All images were captured from the inferior retina, which comprises primarily S-cones. INL, inner nuclear layer; IS, inner segments; ONL, outer nuclear layer. Scale bar: 20 μm.

In WT mouse retina, CK-MT1A was present in rod and cone inner segments, in cone but not rod somas in the outer nuclear layer, and in photoreceptor terminals in the outer plexiform layer (Figures 9A–C). The pattern of expression at P7 was similar to WT (Figures 9D–F). By P14, rod death is quite advanced and CK-MT1A colocalizes with S-opsin in shrunken inner segments, cone somas and axonal terminals (Figures 9G–I). Despite the loss of inner segments from P21, surviving cone somas and axonal terminals in the *rd1* retina were strongly positive for CK-MT1A at all time points examined, encompassing P7 to P60 (Figures 9J–R). Labeling of cone cell bodies for CK-MT1A was considerably stronger than at P7 or in WT retinas.

**FIGURE 10:**
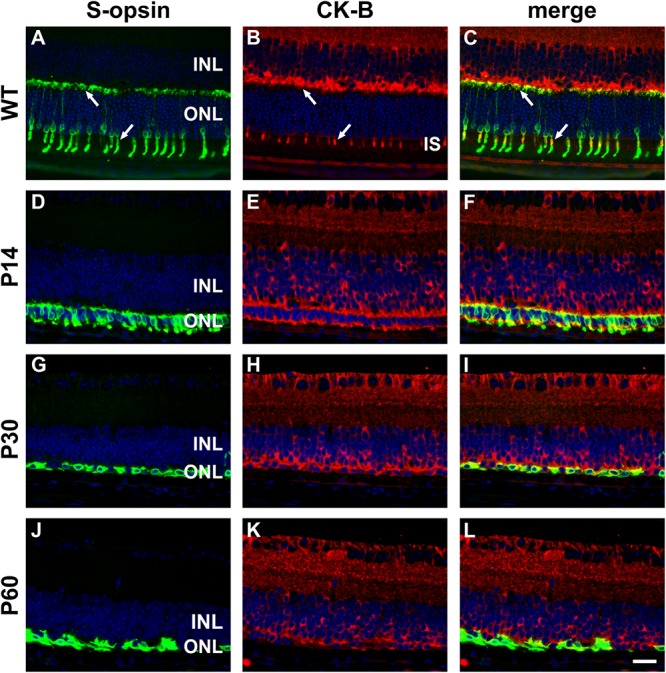
Representative double labeling immunofluorescence images of cytosolic creatine kinase (CK-B) with S-opsin in wild-type (WT) mouse retina and in *Rd1* mouse retinas from postnatal day (P) 14 to P60. In WT retinas **(A–C)**, CK-B colocalizes with S-opsin in cone inner segments (arrows), and in axonal terminals in the outer plexiform layer (arrows). At P14, CK-B colocalizes with S-opsin in shrunken inner segments and axonal terminals, with weak CK-B labeling of somas **(D–F)**. From P30 to P60, the ONL comprises a single layer of surviving cones largely devoid of segments. CK-B colocalizes with S-opsin in cone somas and axonal terminals **(G–L)**. At all time points, CK-B is also expressed by inner retinal neurons and Müller cells. All images were captured from the inferior retina, which comprises primarily S-cones. INL, inner nuclear layer; IS, inner segments; ONL, outer nuclear layer. Scale bar: 20 μm.

In WT mouse retina, CK-B was present in cone but not rod inner segments and in terminals in the outer plexiform layer (Figures 10A–C). From these results, it might be inferred that creatine kinase plays a greater role in cones compared with rods. As for CK-MT1A, surviving cones in the *rd1* retina were positive for CK-B at all time points examined, encompassing P7 to P60 (see Figures 10D–L). Unlike the WT retina, in which cone somal labeling of CK-B was undetectable, from P21 onward cone cell bodies were clearly positive for CK-B.

### Dietary Creatine Supplementation Augments Cone Survival in *Rd1* Retinas

**FIGURE 11:**
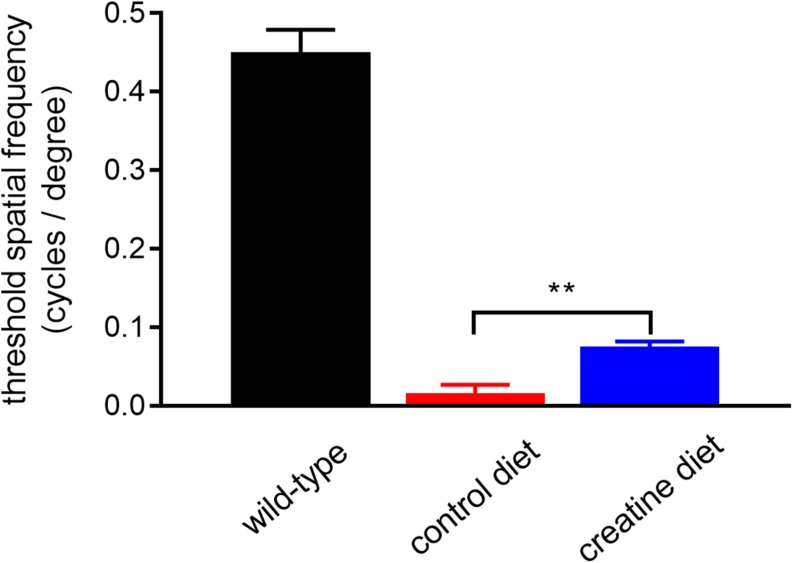
Performance of *Rd1* mice in the optomotor tracking test undertaken at P30. Values represent mean ± SEM, where *n* = 18–21. ^∗∗^*P* < 0.01 by Student’s unpaired *t*-test (creatine-enriched diet vs. control diet eyes).

**FIGURE 12:**
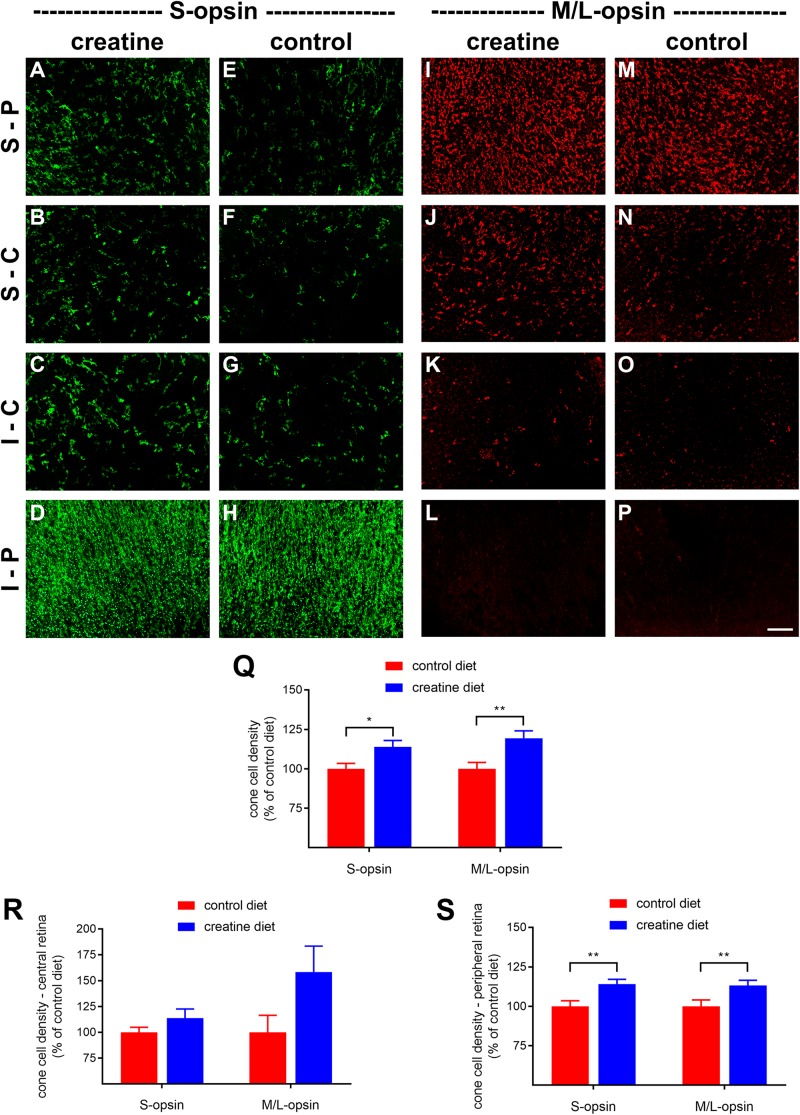
Effect of creatine supplementation on cone survival. **(A–P)** Photomicrographs of wholemount *Rd1* retinas analyzed at postnatal day (P) 60 immunolabeled for S-opsin and M/L-opsin. Representative images from mice fed a creatine-enriched diet **(A–D,I–L)** or a normal diet **(E–H,M–P)** are shown. S-P, superior-peripheral retina; S-C, superior-central retina; I-C, inferior-central retina; I-P, inferior-peripheral retina. Scale bar 100 μm. **(Q–S)** Quantification of S-opsin-labeled cones and M/L-opsin-labeled cones in retinal wholemounts from *Rd1* mice fed a creatine-enriched diet or a standard diet. Data are shown for the whole retina **(Q),** subdivided into central **(R)**, and peripheral **(S)** regions. Values, shown as % of control diet, represent mean ± SEM, where *n* = 18–21. ^∗^*P* < 0.05, ^∗∗^*P* < 0.01 by Student’s unpaired *t*-test (creatine vs. control eyes).

*rd1* mice received either normal chow or 2% oral creatine diet starting at P21. Optomotor testing was performed at P30, the results of which revealed that the creatine-enriched group exhibited approximately 4.5-fold greater visual acuity compared to the control diet cohort (*P* < 0.01, by unpaired Student’s *t*-test; Figure 11). At P60, the number of surviving cones were quantified. Figures 12A–P shows representative photomicrographs of S-opsin^+^ and M/L-opsin^+^ cones from each treatment group. Quantification of the density of S-opsin^+^ and M/L-opsin^+^ cones in the whole retina revealed a modest, but statistically significant, protection of S-opsin^+^ and M/L-opsin^+^ cones in the creatine group compared to the normal diet cohort (*P* < 0.05; *P* < 0.01, respectively, by Student’s unpaired *t*-test; Figure 12Q). When the data were subdivided into central (Figure 12R) and peripheral (Figure 12S) regions, the same patterns were evident, but the neuroprotective effect of creatine only reached significance in the peripheral retina for each cone type (*P* < 0.01, by Student’s unpaired *t*-test).

## Discussion

Targeting secondary cone degeneration is a broad-spectrum strategy applicable to a large proportion of RP subtypes irrespective of the primary gene defect. An increasing body of evidence has implicated energetic insufficiency as a key component of cone degeneration, and, in the present study, we utilized *in vitro* and *in vivo* models of RP to explore whether increasing the availability of two nutrients promotes cone survival. Our results showed that glucose improved cone survival in retinal cultures subjected to mitochondrial stress or oxidative insult; however, daily subconjunctival injections of glucose neither enhanced spatial visual performance nor slowed cone cell degeneration in *rd1* mice relative to isotonic saline. In contrast, creatine not only promoted cone survival in retinal cultures, but also improved vision and reduced cone degeneration in *rd1* mice. The results of this study provide tentative support for the hypothesis that creatine supplementation may delay secondary degeneration of cones in individuals with RP.

### Glucose Supplementation for Cone Survival

In order to mimic cone loss *in vitro* as a first step to understanding the potential role of nutraceutical protection of cones *in situ*, we established culture preparations in which cones were present. Immunocytochemical labeling of these cultures revealed a distinct population of small cells with dendrites, but no obvious segments. In fact, photoreceptors within the culture system employed lose their segments during the first 24 h *in vitro*. The absence of outer segments from otherwise viable photoreceptor cells in similar mixed cultures has been described previously (Gaudin et al., 1996). Our culture system can thus be used to assess the survival of cones whose segments have degenerated or are in the process of doing so. This model, therefore, is of direct relevance to RP, since degeneration of segments is an early pathological event in RP.

We subjected cultures to metabolic disturbance (sodium azide) and oxidative stress (tbH), each of which is implicated in the pathology of cone loss in RP. In both scenarios, concentration-dependent toxicity to S-opsin^+^-cones was noted that was counteracted by pretreatment with glucose. Glucose has been shown to protect against retinal cell loss induced by azide in a previous *in vitro* study (Wood et al., 2012). Notably, however, protection was greater to glia than inner retinal neurons, a finding explained by the greater reliance of neurons on mitochondrial metabolism. The degree of cone protection was similar to that measured for inner retinal neurons previously, implying that the metabolism, and mitochondrial reliance of these cell types is analogous. It may seem that enhancing glycolysis by supplementation with glucose would inevitably counteract mitochondrial compromise, but this makes the assumption that cell metabolism is plastic and can respond rapidly and efficiently to altered energy demands and changing substrate supplies. In the present study, cells are maintained with pyruvate and glutamine, both of which are predominantly metabolized via mitochondria-based pathways. When mitochondria are made dysfunctional with azide, cells will die unless aerobic glycolysis can be alternatively promoted; the addition of glucose offers that possibility, but relies on the metabolic flexibility of the cells. Since aerobic glycolysis contributes to photoreceptor metabolism *in situ* (Chinchore et al., 2017), it is unsurprising that cones can respond to mitochondrial disturbance *in vitro*.

The protective effect of glucose against induced oxidative stress is highly unlikely to be mediated through a direct antioxidative action. Instead, glucose is able to counteract excessive oxidative stress by activation of the pentose phosphate pathway, which produces NADPH to maintain cellular glutathione levels (Ben-Yoseph et al., 1996). Glutathione is the major intracellular antioxidant and maintenance of its active reduced state occurs via the enzyme glutathione reductase. Expression of this enzyme has been demonstrated in photoreceptors *in situ* (Fujii et al., 2001). This, then, is a key action of glucose: maintaining control over oxidative attack via stimulation of additional or alternate metabolic pathways.

Healthy photoreceptors display a high rate of aerobic glycolysis (Chinchore et al., 2017). Accordingly, they express a specific complement of glycolytic isoenzymes, including hexokinase II, pyruvate kinase M2, and LDH-V, which are concentrated in their inner segments (Casson et al., 2016; Petit et al., 2018). The importance of hexokinase II for cone homeostasis has recently been established in a study by Petit et al. (2018), who showed that deletion of HK2 has minimal impact upon cone viability in normal conditions, but, that HK2 confers a survival advantage in cones under conditions of metabolic stress. Since loss of cone segments is an early pathological event in RP, we investigated whether surviving cones in the *Rd1* retina continue to express genes vital for aerobic glycolysis. The data showed that, at all ages analyzed, cone somas in the *Rd1* retina expressed hexokinase II and LDH-A (as well as PKM2 and neuron-specific enolase). The robust labeling of hexokinase II and LDH-A in surviving cones testifies to the fact that the biochemical machinery required for aerobic glycolysis persists despite the irrevocable disruption to their normal cellular architecture. Boosting energy supplies should allow cones to survive for longer and may even facilitate the process of segment regeneration, in an analogous manner to that observed in experimental retinal detachment following oxygen supplementation (Mervin et al., 1999).

The lack of protective effect of local glucose delivery in *Rd1* mice may be due to a number of factors. The first issue to consider is how much additional glucose is available for uptake by cones. Subconjunctival injection is considerably less invasive than intravitreal or subretinal injections, permitting daily administration, and resulting in higher bioavailability than other periocular injection sites; however, retinal bioavailability is lower than intravitreal injection owing to the various barriers between the sites of administration and the target (Del Amo et al., 2017). In previous work, we showed that subconjunctival injection of glucose elevated the vitreal glucose level and protected retinal ganglion cells from ischemia-reperfusion (Shibeeb et al., 2016), while in the present study, a single subconjunctival injection of glucose resulted in >threefold elevation of the vitreous glucose concentration. These data suggest that subconjunctivally administered glucose likely reaches the photoreceptor layer; however, it should be recognized that our earlier study featured target neurons that reside closest to the vitreous humor, as well as an acute, rather than chronic, model of injury. In the *Rd1* mouse, inner retinal neurons and glia would be exposed to the glucose before it reaches the cones, reducing the total amount delivered. To shed some light on this question, we analyzed tissue sections of retina from wild type mouse eyes that had undergone a subconjunctival injection of 2-NDBG, a stable, fluorescent glucose derivative used for monitoring glucose uptake into living cells. The data showed 2-NBDG fluorescence within photoreceptor inner and outer segments, confirming that at least some glucose reaches the photoreceptor layer after subconjunctival injection. The pattern of 2-NBDG fluorescence was similar to that reported after oral administration of the drug in mice (Kanow et al., 2017). While these qualitative data verify that glucose reaches cones after subconjunctival injection, they do not reveal the concentration.

A second issue relates to timing. Whilst there was a substantial increase in vitreal glucose after 1 h, the concentration decreased quite rapidly thereafter. In a chronic model of degeneration, controlled, slow release of drug is advantageous to ensure continued bioavailability. In contrast to the subconjunctival route, subretinal injection delivers glucose directly to cones, and excitingly this methodology has been shown to cause a short-term improvement in cone function as well as outer segment synthesis when tested in the early stages of cone pathology in the P23H porcine model of RP (Wang et al., 2016). Subretinal injection is, however, technically challenging and only suited to one-off applications such as delivery of genes or stem cells (Del Amo et al., 2017).

An additional explanation for the lack of efficacy of glucose on cone survival relates to reduced uptake of glucose into cones. Extracellular glucose is believed to enter cones primarily via GLUT1, although other uptake mechanisms may exist since GLUT1 expression in cones appears to be very modest given the extraordinarily high metabolic activity of these cells (Gospe et al., 2010; Ait-Ali et al., 2015; Swarup et al., 2019). The activity of GLUT1 is stimulated by a soluble factor released from rods (Ait-Ali et al., 2015). In *rd1* mice, rod death will result in less efficient uptake of glucose into cones and reduced aerobic glycolysis. In response, cones compensate by increasing synthesis of GLUT1 in a bid to increase intracellular glucose (Punzo et al., 2009; Venkatesh et al., 2015). Thus, although more glucose may be available for uptake after local delivery of glucose, this may not translate to a higher intracellular concentration. Therapies that target GLUT1 or its binding partner BSG-1 may need to be employed (Ait-Ali et al., 2015). A final point worth making is that, in our study, glucose supplementation began at P14 when rod photoreceptors would still be present. It is possible that any effects of glucose on cones were influenced by the presence of rods. Glucose may conceivably have influenced the time course of rod degeneration, but since retinas were not analyzed until P60, this data is not available.

The finding that saline injections had a small beneficial effect on visual function and cone survival was not entirely unexpected. It has been proven that saline or dry needle injections into the subretinal space provide a modest, but significant, boost to photoreceptor survival in inherited or induced models of degeneration via a mechanism of action hypothesized to involve the release of survival factors (Faktorovich et al., 1990, 1992; Silverman and Hughes, 1990). It is likely that repeated subconjunctival injections elicited an analogous response.

### Creatine Supplementation for Cone Survival

Creatine supplementation has been shown to be support neuronal survival in a variety of animal models of neurodegenerative disease, including Alzheimer’s disease (Brewer and Wallimann, 2000), Parkinson’s disease (Matthews et al., 1999), amyotrophic lateral sclerosis (Klivenyi et al., 1999), and Huntington’s disease (Matthews et al., 1998; Ferrante et al., 2000; Andreassen et al., 2001). In the present study, creatine supplementation protected cones against mitochondrial compromise *in vitro*, and, improved visual function and cone survival in *rd1* mice.

The first important finding was that surviving cone photoreceptors in *rd1* mice continue to express creatine kinase. Despite the loss of their mitochondrial-rich inner segments, cone somas and axonal terminals in the *rd1* retina were strongly positive for both the mitochondrial and cytosolic forms of creatine kinase at each time point examined. The data reveal that the fundamental enzymes of the creatine-phosphocreatine system are preserved in surviving cones of the *rd1* retina and attest to the logic of the neuroprotective strategy.

The cone protection afforded by creatine has several possible mechanisms. Exogenous creatine supplementation has been shown to increase the total intracellular phosphocreatine level (Beal, 2011). Phosphocreatine provides energy to photoreceptor outer segments for phototransduction and to synaptic terminals for neurotransmission (Linton et al., 2010; Hurley et al., 2015). Hence, creatine-induced cone preservation may simply result from increasing the available phosphocreatine pool, thereby supporting overall cellular energy production. Secondly, and relatedly, mitochondrial creatine kinase is tightly coupled to ATP synthesis, respiratory chain activity and ATP export. This coupling improves the efficiency of oxidative phosphorylation, and the transport and utilization of intracellular energy. Creatine supplementation is believed to enhance these functions through the action of creatine kinase (see Wallimann et al., 2011). In the culture experiments outlined in the present study, creatine was clearly protective against cone loss induced by mitochondrial inhibition; this action could be explained by either of the above mechanisms. Thirdly, numerous studies have shown that exogenous creatine is highly effective at reducing cellular damage caused by oxidative stress (see Wallimann et al., 2011). This has been largely explained by the fact that creatine kinase stabilizes mitochondrial membranes, reducing the release of reactive oxygen species, although creatine is also a weak free radical scavenger itself and has been shown to protect against oxidative stress directly (Lawler et al., 2002). In the present study, however, creatine was not able to protect cones in culture from the effects of the tbH, thus dismissing the possibility of it having a direct antioxidant mode of action *in vitro*. This does not discount the possibility that creatine can act in an indirect antioxidant capacity *in situ*, however, via a reduction in mitochondrial release of free radicals. If this were the case, then this is highly relevant to RP since an increasing body of evidence implicates oxidative stress in the pathogenesis of cone degeneration in the disease (Shen et al., 2005; Komeima et al., 2006, 2007; Usui et al., 2009; Lee et al., 2011; Campochiaro et al., 2015). Finally, creatine displays anti-apoptotic properties, by virtue of inhibiting opening of the mitochondrial permeability transition pore, and induction of differential expression of pro-survival transcription factors and cell signaling pathways (see Wallimann et al., 2011). Further investigation is needed to definitively establish the precise mechanisms by which creatine enhances cone survival in the *rd1* mouse.

The major limitation of the present study is the early onset, and fast progression, of cone degeneration in the *rd1* model of RP. Creatine supplementation was initiated at the time of weaning, P21. By this age, however, all rod constituents and the majority of cone outer segments had degenerated. *rd1* mice do not produce a recordable electroretinogram for many days beyond P21 (Farber et al., 1994). The improvement observed in the optokinetic response in creatine-fed mice suggests that clinically useful visual function might be preserved even when cone degeneration has progressed to a late stage. Nevertheless, the use of a slower progressing model of degeneration, such as the *rd1*0 strain, would permit more expansive evaluation of cone structure and function, including quantification of the rate of cone segment loss, and measurement of the electroretinogram (Wang et al., 2014).

The results of randomized clinical trials with oral creatine supplementation for neurodegenerative diseases have largely been disheartening to date. A number of clinical trials investigating the efficacy of creatine in Parkinson’s disease all failed to meet primary end points, despite some evidence of improvements in secondary outcomes (Bender and Klopstock, 2016). Likewise, clinical outcomes in trials of creatine in Huntingdon’s disease and Amyotrophic lateral sclerosis have also been disappointing (Bender and Klopstock, 2016). Nevertheless, there are convincing reasons for further investigation of creatine in RP: firstly, creatine kinase is extremely abundant in mammalian photoreceptors and appears to play a pivotal role in vision (Linton et al., 2010); secondly, RP can be diagnosed prior to secondary cone degeneration. From the credible results of preclinical studies, it has been argued that prophylactic treatment with creatine to prevent neuronal degeneration is far more effective than treatment at later disease stages; thirdly, there are no current therapies for RP and any improvements in visual quality would be beneficial for individuals; fourthly, the safety and ease of delivery of creatine supplementation has been established and is clinically appealing, although it must be recognized that adverse effects such as gastrointestinal complaints, muscle cramps and an increase in body weight have been reported to occur occasionally after creatine supplementation (Andres et al., 2017). The development of a targeted ocular delivery system, which bypasses the systemic circulation, would potentially provide a safe, long-term route of administration of even higher doses of creatine, which has shown promise in individuals with premanifest Huntingdon’s disease (Rosas et al., 2014).

## Data Availability Statement

The datasets generated for this study are available on request to the corresponding author.

## Ethics Statement

The animal study was reviewed and approved by the Animal Ethics Committees of SA Pathology, Central Adelaide Local Health Network (CALHN) and the University of Adelaide (Adelaide, SA, Australia) and conformed with the Australian Code of Practice for the Care and Use of Animals for Scientific Purposes, 2013, and with the ARVO Statement for the use of animals in vision and ophthalmic research.

## Author Contributions

All authors: full access to all the data in the study, take responsibility for the integrity of the data, and the accuracy of the data analysis. RC and GC: study concept and design. DN, GC, and JW: acquisition of data, and analysis and interpretation of data. DN, RC, and GC: drafting of the manuscript. JW: critical revision of the manuscript for important intellectual content. DN and RC: statistical analysis. RC: obtained funding, administrative, technical, and material support.

## Conflict of Interest

The authors declare that the research was conducted in the absence of any commercial or financial relationships that could be construed as a potential conflict of interest.
