# Supplementary material for: Investigations Into Bioenergetic Neuroprotection of Cone Photoreceptors: Relevance to Retinitis Pigmentosa
Source: Front Neurosci. 2019 Nov 15;13:1234. doi: 10.3389/fnins.2019.01234 (PMC6872495; doi:10.3389/fnins.2019.01234)
Supplement: Supplementary file 1 [file Data_Sheet_1.DOC]

**A** **B**


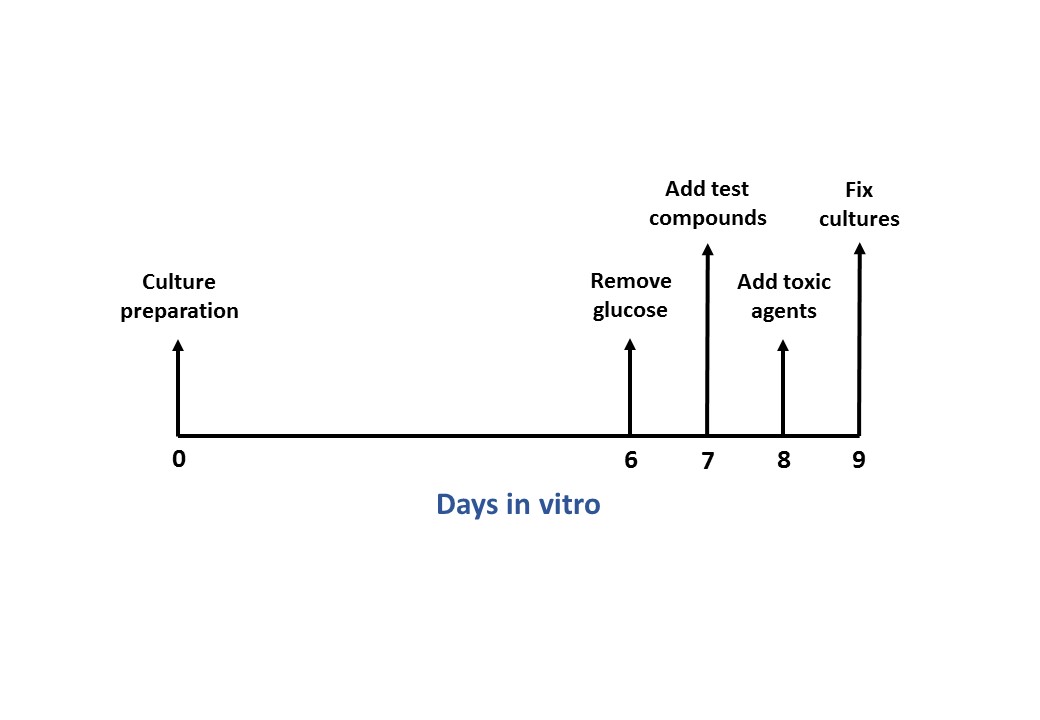

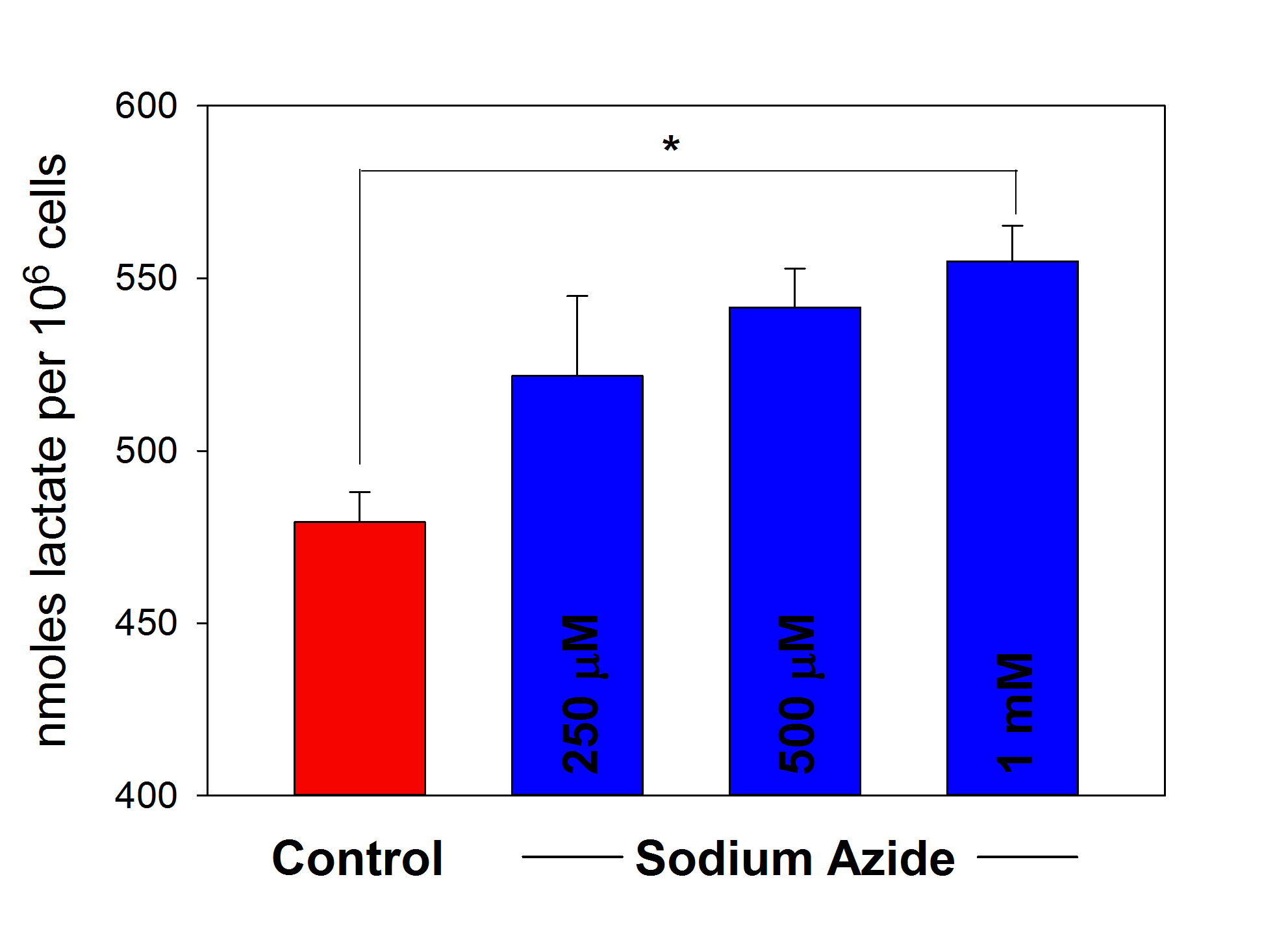


**Supplementary Figure S1**. (**A**) Schematic representing the timeline of culture experiments. (**B**) Effect of various concentrations of sodium azide on lactate production in mixed retinal cultures over a period of three hours. Data represent mean±SEM. P<0.05 by ANOVA, followed by Dunnett’s multiple comparisons test.
